# Cytokine Profiles at Admission Can Be Related to Outcome in AIDS Patients with Cryptococcal Meningitis

**DOI:** 10.1371/journal.pone.0120297

**Published:** 2015-03-23

**Authors:** Delio José Mora, Laila Rigolin Fortunato, Leonardo Eurípedes Andrade-Silva, Kennio Ferreira-Paim, Ivonete Helena Rocha, Rakel Rocha Vasconcelos, David Nascimento Silva-Teixeira, Gabriel Antonio Nogueira Nascentes, Mario León Silva-Vergara

**Affiliations:** 1 Infectious Diseases Unit, Internal Medicine Department, Triângulo Mineiro Federal University, Uberaba, Minas Gerais, Brazil; 2 Federal Institute of Triângulo Mineiro, Uberaba, Minas Gerais, Brazil; University of Birmingham, UNITED KINGDOM

## Abstract

Cryptococcal meningitis (CM) remains as common life-threatening AIDS-defining illness mainly in resource-limited settings. Previous reports suggested that baseline cytokine profiles can be associated to fungal burden and clinical outcome. This study aimed to evaluate the baseline cytokine profiles in AIDS patients with CM and its relation with the outcome at weeks 2 and 10. Thirty AIDS patients with CM diagnosed by cerebrospinal fluid (CSF) *Cryptococcus neoformans* positive culture, India ink stain and cryptococcal antigen test were prospectively evaluated. As controls, 56 HIV-infected patients without CM and 48 non-HIV individuals were included. Baseline CSF and sera levels of IL-2, IL-4, IL-8, IL-10, IL-12p40, IL-17A, INF-γ and TNF-α were measured by ELISA. Of 30 CM patients, 24 (80%) were male, median age of 38.1. The baseline CSF high fungal burden and positive blood culture were associated with a positive CSF culture at week 2 (p = 0.043 and 0.029). Most CSF and sera cytokines presented higher levels in CM patients than control subjects (p < 0.05). CSF levels of IL-8, IL-12p40, IL-17A, TNF-α, INF-γ and sera TNF-α were significantly higher among survivors at weeks 2 and 10 (p < 0.05). Patients with increased intracranial pression exhibited CSF IL-10 high levels and poor outcome at week 10 (p = 0.032). Otherwise, baseline CSF log10 IFN-γ and IL-17A were negatively correlated with fungal burden (r = -0.47 and -0.50; p = 0.0175 and 0.0094, respectively). The mortality rate was 33% (10/30) at week 2 and 57% (17/30) at week 10. The severity of CM and the advanced immunodeficiency at admission were related to a poor outcome in these patients. Otherwise, the predominant Th1 cytokines profile among survivors confirms its pivotal role to infection control and would be a prognostic marker in cryptococcal meningitis.

## Introduction

Cryptococcal meningitis (CM) is a common life-threatening fungal infection in AIDS patients and represents a medical, social and economic high burden due to its unacceptable 60% mortality rate [1,2]. This occurs particularly in poor-resources settings where most individuals present late HIV diagnosis and severe fungal disease at admission together with a limited access to anti-retroviral (ART) and ideal antifungal therapy [1–3]. Otherwise, in high-income countries, CM number of cases and mortality rate declined significantly following introduction of more effective ART and a gold standard antifungal therapy [4,5].

Yearly, at least one million of cryptococcosis cases occur around the world, mostly in HIV infected individuals of whom 620,000 die, predominantly in sub-Saharan Africa where the mortality overpasses that associated to tuberculosis despite the expansion of ART programs and FLZ availability during the last years [2,6]. Latin America is the third global region with high incidence and a 10-week mortality rate of 30 to 55% [7–9].

In order to understand the poor outcome of CM patients, several clinical and laboratory features have been evaluated as prognostic markers such as: to be naive to ART, altered consciousness, abnormal brain imaging at computerized tomography (CT) scan and/or magnetic resonance imaging (MRI), elevated intracranial pressure (ICP) and disseminated infection at admission [3,10–13]. Furthermore, CD4^+^ T count < 100 cells/mm^3^, CSF white blood cell (WBC) count ≤ 20 cells/μL, CSF culture >10^6^ CFU/mL with positive India ink stain, paucity of Th1 pattern cytokine release and infection with *C*. *neoformans* var. *grubii* (serotype A) have been also considered [14–16].

Similar to other chronic fungal and mycobacterial diseases, cryptococcal infection clearance is associated with granulomatous inflammatory reaction and depends on coordinated interaction of innate and adaptive immune response mediated by CD4^+^ and CD8^+^ T-cells to establish a type 1 helper T cells (Th1) response [17–19]. This protective immune response involves the releasing of cytokines such as tumor necrosis factor α (TNF-α), interleukin 8 (IL-8), IL-6, IL-12 and interferon γ (IFN-γ) which lead to classical activation of macrophages and their recruitment to the infection site [14,19,20].

In contrast, high levels of IL-4, IL-5, IL-10 and IL-13 have been related to inadequate IFN-γ production and alternatively activation of macrophages leading to uncontrolled fungal infection [21–23]. This fact has been attributed to immune modulatory properties of capsular cryptococcal antigen (CrAg) continuously released favoring the yeast escape from the immune system [24,25]. Moreover, it has been associated with raised ICP, immune reconstitution inflammatory syndrome (IRIS), disease severity and a poor outcome [26–28]. Of note, in animal models, IL-17A high levels were associated with reduced fungal burden and a protective immune response against pulmonary infection with *C*. *neoformans* H99γ [29]. However the role of these cytokines in patients with cryptococcal infection is yet to be elucidated [30].

This study aimed to evaluate the baseline CSF and sera cytokines and their relation with clinical and laboratory features and outcome in AIDS patients with cryptococcal meningitis.

## Methods

### Subjects

This study was carried out from August 2008 to November 2012 at the Teaching Hospital of Triângulo Mineiro Federal University in Uberaba, Minas Gerais State, Brazil. Thirty out of 38 AIDS-patients aged ≥ 18 years presenting CM (CM^+^ HIV^+^) were prospectively enrolled. Cryptococcal meningitis case was defined on clinical and laboratory features including positive CSF India ink stain, CrAg test and *Cryptococcus neoformans* culture [31]. Demographic, epidemiological, clinical, laboratory and outcome data were obtained from their medical records. As controls to CSF and sera baseline cytokines assessment, 56 HIV-positive individuals without CM (CM^-^ HIV^+^) matched by CD4^+^ T-cells count, age and gender admitted at the hospital due to several infectious such as: toxoplasmosis, chagas disease, syphilis, tuberculosis, cytomegalovirus (CMV) and *Paracoccidioides brasiliensis* or non-infectious neurological clinical pictures such as: epilepsy, migraine, stroke, dementia, tension-type headache, among others were included. These patients required lumbar puncture for elucidative clinical diagnosis and a CSF aliquot was collected for this study. Otherwise, another control group was formed by 48 HIV-negative patients without cryptococcosis (CM^-^ HIV^-^) admitted to the emergency room for several reasons such as: migraine, epilepsy, tension-type headache, stroke, dementia, among others were included. During clinical evaluation, these patients performed lumbar puncture and a CSF aliquot was collected for this study (Table 1).

**Table 1:** Demographic, clinical and laboratory data of patients with cryptococcal meningitis and the control groups.

Variables	CM^+^ HIV^+^	CM^-^ HIV^+^	CM^-^ HIV^-^
	N = 30	N = 56	N = 48
Age, years	38.1 (25–50)	38.4 (31–44)	40.1 (33–47)
Males N (%)	24 (80)	37 (66)	38 (79.1)
Years since HIV diagnosis	12.8 (9.3–18.4)	10.5 (7.1–17.8)	——
CD4^+^ T-cells count, cells/mm^3^	36 (19–75)	39.4 (25–54)	——
HIV load, log_10_ copies/mL	5.1 (4.3–5.9)	5 (5–5.1)	——

Data presented are median [interquartile range, (IQR)] or percentage (No.). Groups are comparable in CD4^+^ T-cell count and HIV load in blood (p = 0.027 and 0.042, respectively).

Abreviations: CM^+^ HIV^+^, HIV-infected patients with cryptococcal meningitis; CM^-^ HIV^+^, HIV-infected individuals without cryptococcal meningitis; CM^-^ HIV^-^, HIV-negative individuals without cryptococcosis.

**CM^-^ HIV^+^ control group clinical diagnoses**: syphilis (n = 7), encephalitis associated to HIV infection (n = 7), toxoplasmosis (n = 5), tension-type headache (n = 5), dementia associated to HIV (n = 5), asseptic meningitis (n = 4), progressive multifocal leucoencephalopaty (n = 3), *mycobacterium* disease (n = 2), viral encephalitis (n = 2), epilepsy (n = 2), cerebral primary lymphoma (n = 2), migraine (n = 2), stroke (n = 1), encephalitis by *T*. *cruzi* (n = 1), encephalitis by *Paracoccidioides brasiliensis* (n = 1), cerebral nocardiosis (n = 1), guillain-barré syndrome (n = 1), encephalitis by CMV (n = 1), sepsis (n = 1), glaucoma (n = 1), hypertensive encephalopaty (n = 1), unknown paresis (n = 1).

**CM^-^ HIV^-^ control group clinical diagnoses**: epilepsy (n = 10), migraine (n = 7), stroke (n = 6), tension-type headache (n = 6), skull trauma (n = 6), alcoholism (n = 5), dementia (n = 2), systemic erithematous lupus (n = 1), hipertensive encephalopathy (n = 1), asseptic meningitis (n = 1), viral encephalitis (n = 1).

The HIV infection status was defined by two enzyme-linked immunoassay tests (ELISA) (Welcozyme, Wellcome diagnostics, Dart ford, Oxford, UK) and confirmed by Western blot. Written informed consent of patients and controls was provided. The study was approved by the Research Ethical Board of the Triângulo Mineiro Federal University (protocol #1350).

### Laboratory assessment

Both blood and CSF samples of CM patients and controls were simultaneously obtained at admission. The CSF samples (5–10 mL) were divided in two parts; the first was used to perform cell count, biochemical assessment, cryptococcal antigen (CrAg) detection and quantitative fungal culture. The second was aliquoted (200μL/vial) and stored at -70°C to cytokine assessment. The blood samples (5–10 mL) were centrifuged and sera was aliquoted (200μL/vial) and stored at -70°C to further cytokine assessment and capsular antigen quantification. The CSF and sera CrAg titers were determined by the cryptococcal antigen latex detection system (IMMY Mycologics Inc, OK, USA) according to manufacturer’s protocol. This semiquantitative technique to determine the CrAg titer was based on the agglutination observed in serial dilutions of CSF and sera. The fungal identification included the presence of capsule by India ink stain, ability to produce melanin on Niger seed (*Guizotia abyssinica*) agar and urease production (Urease Christensen). L-Canavanine-glycine-bromothymol blue (CGB) agar and orotidine monophosphate pyrophosphorylase (*URA5*) gene restriction fragment length polymorphism (RFLP) analysis were used to identify *Cryptococcus* species and its genotype, respectively according to methodology already described [32,33].

Quantitative fungal culture was performed as described elsewhere [31]. Briefly, with a mean delay of < 1 h after lumbar puncture, 1 mL of CSF was diluted 10-fold in sterile saline and 100μL of each dilution were inoculated onto Sabouraud’s dextrose agar (SDA) plate (DIFCO, Detroit, Michigan). Plates were incubated at 30°C for 14 days and colonies counted at the lowest dilution to evidence discernible colonies which multiplied by the dilution, permits to obtain colony-forming units (CFU) per mL and then log_10_-transformed.

### Cytokine assays

Sera and CSF samples of CM patients and control subjects stored at -70°C were used to evaluate cytokines levels through Enzime-Linked Immunosorbent Assay (ELISA). Tumor necrosis factor-α (TNF-α), Interferon-γ (INF-γ), Interleukin-2 (IL-2), IL-4, IL-8, IL-10, IL-12p40, (Becton Dickinson, USA) and IL-17A (R&D Systems, USA) levels were quantified using the luminometer (Turner Biosystems, Sunnyvale, CA, USA). Briefly, 96-well half area flat-bottomed plates (Costar, Corning, NY) were prepared by coating the wells overnight at 4°C with 35μL of a Capture Antibody Purified Anti-human for each cytokine in Coating Buffer (1:250). Each well was washed 5 times (150 μL/well) with PBS-Tween wash solution (PBS/0.05% Tween 20) and then blocked with 100μL/well of PBS/2% BSA for 4 h at room temperature. After well washing for 5 times with wash solution, 25 μL of test samples diluted in assay buffer (25μL PBS/1% BSA) or cytokine standards (serial dilution 1:2) were added to washed wells and incubated overnight at 4°C. Washed wells were incubated with 35μL of Detection Antibody Biotin Anti-human for each cytokine (1:500) and Enzyme Concentrate Streptavidin-HRP (1:250) in assay buffer (PBS/1% BSA) for 2 h at 37°C. The plates were again washed five times and added 50 μL of freshly mixed TMB peroxidase solutions A and B (1:1 [vol/vol]) per well at room temperature. The reaction was stopped after 5 min by the addition of 50 μL of 1 M sulfuric acid per well and the color absorbance was read at OD 405 and 490 nm (Abs405—Abs490). The results were expressed in picograms per milliliter based on a standard curve.

### Statistical analysis

Continuous variables with normal distribution were analyzed by Student’s t-test, and continuous variables with abnormal distribution were analyzed by the Mann-Whitney U-test. Correlations to continuous variables were tested for significance through the Spearman rank and Pearson’s tests. Cytokine median levels were compared by the Mann-Whitney U-test or Kruskal-Wallis test with Dunn's multiple, depending on the groups’ number. The association between baseline cytokine levels and CrAg titers with mortality at weeks 2 and 10 was evaluated by Mann-Whitney U test. The Kaplan-Meier method and the log-rank test were used to evaluate the cumulative survival by univariate analysis at weeks 2 and 10 according to clinical and laboratory parameters. Factors significantly associated with risk of death by univariate analysis underwent a multivariate analysis using Cox proportional-hazards regression models to identify parameters that were independently related to mortality. The Hazard ratio (HR) was estimated by the uni- and mutivariate analysis with 95% confidence interval to show the effect of each variable on the death risk at weeks 2 and 10. Statistical analyses were performed using SPSS 17.0 (SPSS, Chicago, IL, 2008), MedCalc for Windows version 11.3 (MedCalc Software, Ostend, Belgium) and GraphPad Prism v5 (GraphPad software Inc, CA USA). For all tests, p values < 0.05 were considered significant.

## Results

### Baseline epidemiological, clinical and laboratory data

Of 30 AIDS-patients with CM, 24 (80%) were male, mean age of 38.1 years (interquartile range [IQR, 25–50]). Cryptococcal meningitis was the first AIDS-defining disease in 17 (56.6%) cases, while in 11 (64.7%) both diagnoses were simultaneously performed at admission. The median CD4^+^ cell count was 36/mm^3^ [IQR, 19–75] and the median HIV load was 5.1 log_10_ ARN copies/mL [IQR, 4.3–5.9]. The most prevalent category risk to HIV infection was heterosexual unprotected intercourse present in 26 (86.6%) individuals of whom 13 (50%) were also illicit drug users. All clinical isolates were characterized as *C*. *neoformans*, VNI genotype.

At admission, clinical features such as: headache, fever, stiff neck, weight loss, altered consciousness and increased ICP were present in 26 (86.6%), 23 (76.6%), 22 (73.3%), 22 (73.3%), 19 (63.3%) and 18 (60%) individuals respectively. Mean time between onset of symptoms and hospitalization was 16.2 days (range, 4–27). Analysis of CSF baseline showed ≥ 5 log_10_ CFU/mL, CrAg titer ≥ 1:1024 and WBC count < 20 cells/μL in 20 (66.6%), 20 (66.6%) and 16 (53.3%) of cases, respectively. The mean CSF protein concentration in CM patients (92 mg/dL [IQR, 61–119]) was significantly elevated compared with 34 mg/dL in CM^-^ HIV^+^ and 42 mg/dL in CM^-^ HIV^-^controls (p < 0.001). The mean CSF glucose of (26.4 mg/dL [IQR, 18–50.4]) in CM patients was significantly lower compared with 49.7 mg/dL in CM^-^ HIV^+^ and 51.6 mg/dL in CM^-^ HIV^-^ subjects (p = 0.043). The protein concentration in CM patients was significantly correlated with the CrAg titers (r = 0.78, p = 0.032).

### Clinical and laboratory features vs outcome

Patients with papilledema, weight loss and cranial nerves palsies at admission presented increased risk of death at weeks 2 through univariate analysis (all p ≤ 0.03) whereas, stiff neck, papilledema, weight loss, cranial nerves palsies, increased ICP and cryptococcal meningitis as first AIDS definition disease were associated to a poor outcome at week 10 (all p < 0.05, Table 2). Mortality rate of 33% (10/30) and 57% (17/30) were observed at weeks 2 and 10 on therapy, respectively. Four patients died before antifungal therapy. Among patients with increased ICP, eight (44.4%) performed repeated relief lumbar punctures. Of 26 patients on therapy, 20 (76.9%) received Amphotericin B (AmB) during 14 days and after this fluconazole 400 mg twice a day for eight weeks. The remaining six patients received AmB plus fluconazole for 14 days followed by fluconazole 400 mg twice a day for eight weeks. No cases of immune reconstitution inflammatory syndrome (IRIS) were diagnosed among these patients. Besides, none of them received cortiscosteroid therapy.

**Table 2:** Clinical features at admission associated with outcome at 2 and 10 weeks in 30 AIDS-patients with cryptococcal meninigitis.

Clinical data	2-week mortality			10-week mortality		
	N (%)	p-univariate	HR (95% CI)	N (%)	p-univariate	HR (95% CI)
**Gender**						
** Female**	2/6 (33.3)	0.933	0.94 (0.20–4.41)	3/6 (50.0)	0.824	1.15 (0.33–4.01)
** Male**	8/24 (33.3)			14/24 (58.3)		
**Headache**						
** No**	1/4 (25.0)	0.645	1.62 (0.21–12.84)	2/4 (50.0)	0.610	1.47 (0.34–6.44)
** Yes**	9/26 (34.6)			15/26 (57.7)		
**Fever**						
** No**	2/7 (28.6)	0.827	1.19 (0.25–5.60)	4/7 (57.1)	0.920	1.06 (0.34–3.25)
** Yes**	8/23 (34.8)			13/23 (56.5)		
**Stiff neck**						
** No**	1/8 (12.5)	0.235	3.50 (0.44–27.65)	1/8 (12.5)	**0.040**	8.40 (1.11–63.85)
** Yes**	9/22 (40.9)			16/22 (72.7)		
**Papilledema**						
** No**	2/19 (10.5)	**0.001**	12.77 (2.65–61.45)	8/19 (42.1)	**0.002**	4.86 (1.82–13.01)
** Yes**	8/11 (72.7)			9/11 (81.8)		
**Vomit**						
** No**	5/21 (23.8)	0.083	3.01 (0.87–10.41)	11/21 (52.4)	0.221	1.87 (0.69–5.07)
** Yes**	5/9 (55.6)			6/9 (66.7)		
**Weigth loss**						
** No**	0/8 (0.0)	**0.030**	4.45 (1.16–17.15)	2/8 (25.0)	**0.046**	4.52 (1.03–19.93)
** Yes**	10/22 (45.5)			15/22 (68.2)		
**Altered consciousness**						
** No**	3/11 (27.3)	0.617	1.41 (0.36–5.46)	4/11 (36.4)	0.143	2.32 (0.75–7.15)
** Yes**	7/19 (36.8)			13/19 (68.4)		
**Cranial nerves palsies**						
** No**	3/18 (16.7)	**0.021**	4.98 (1.27–19.43)	6/18 (33.3)	**0.001**	6.01 (2.11–17.14)
** Yes**	7/12 (58.3)			11/12 (91.7)		
**Nausea**						
** No**	9/20 (45.0)	0.118	0.19 (0.02–1.52)	13/20 (65.0)	0.216	0.49 (0.16–1.51)
** Yes**	1/10 (10.0)			4/10 (40.0)		
**Increased ICP**						
** No**	1/12 (8.3)	0.059	7.34 (0.93–58.05)	4/12 (33.3)	**0.032**	3.46 (1.11–10.74)
** Yes**	9/18 (50.0)			13/18 (72.2)		
**Seizures**						
** No**	6/22 (27.3)	0.336	1.86 (0.53–6.60)	10/22 (45.5)	0.069	2.47 (0.93–6.57)
** Yes**	4/8 (50.0)			7/8 (87.5)		
**First AIDS defining disease**						
** No**	2/13 (15.4)	0.079	4.02 (0.85–18.97)	5/13 (38.5)	**0.049**	2.88 (1.01–8.23)
** Yes**	8/17 (47.1)			12/17 (70.6)		

Abbreviations: HR, Hazard ratio; CI, confidence interval; ICP, increased intracranial pressure.

The univariate analysis, showed patients who presented baseline CSF ≥ 5 log_10_ CFU/mL and CrAg titer > 1:1024 had a 5.9-fold increased risk to death at week 2 (p = 0.092). Similarly, patients with CSF ≥ 5 log_10_ CFU/mL, CrAg titer > 1:1024 and fungaemia survived less at week 10 (all p < 0.04, Table 3). Individuals with baseline CSF high fungal burden and positive blood culture presented positive CSF culture at week 2 on therapy (p = 0.043 and 0.029, respectively), while sera CrAg titers were higher in patients with fungaemia (1:2048 vs. 1:64, p < 0.001). The CSF culture was negative in 12/20 (60%) and 11/13 (84.6%) at weeks 2 and 10, respectively. In addition, patients with persistent positive culture at week 2 were also associated to a poor outcome at week 10 (p = 0.039). Otherwise, baseline high fungal burden presented increased risk of death at weeks 2 and 10 (p = 0.023 and 0.034, respectively).

**Table 3:** Laboratory features at admission associated with outcome at 2 and 10 weeks in 30-AIDS patients with cryptococcal meningitis.

Laboratory data	2-week mortality			10-week mortality		
	N (%)	p-univariate	HR (95% CI)	N (%)	p-univariate	HR (95% CI)
**CD4^+^ (cells/mm^3^)**						
**> 100**	3/7 (42.9)	0.613	0.70 (0.18–2.73)	3/7 (42.9)	0.572	1.43 (0.41–5.00)
**≤ 100**	7/23 (30.4)			14/23 (60.9)		
**HIV load (RNA/mL)**						
**< 30.000**	3/7 (42.9)	0.809	0.85 (0.22–3.28)	6/7 (85.7)	0.211	0.53 (0.19–1.44)
**≥ 30.000**	7/23 (30.4)			11/23 (47.8)		
**log**_**10**_ **CFU/mL**						
**< 5 Log**_**10**_	1/10 (10.0)	0.092	5.91 (0.75–46.81)	3/10 (30.0)	**0.041**	3.68 (1.05–12.90)
**≥ 5 Log**_**10**_	9/20 (45.0)			14/20 (70.0)		
**CrAg titer**						
**< 1:1024**	1/10 (10.0)	0.092	5.91 (0.75–46.81)	3/10 (30.0)	**0.041**	3.68 (1.05–12.90)
**≥ 1:1024**	9/20 (45.0)			14/20 (70.0)		
**CSF-WBC count**						
**≥ 20 cells/μL**	2/14 (14.3)	0.058	4.49 (0.95–21.24)	6/14 (42.9)	0.088	2.39 (0.88–6.51)
**< 20 cells/μL**	8/16 (50.0)			11/16 (68.8)		
**CSF glucose**						
**≥ 50 mg/dL**	3/8 (37.5)	0.840	0.87 (0.22–3.37)	3/8 (37.5)	0.338	1.84 (0.53–6.42)
**≤ 50 mg/dL**	7/22 (31.8)			14/22 (63.6)		
**CSF protein**						
**≤ 40 mg/dL**	2/8 (25.0)	0.550	1.60 (0.34–7.56)	3/8 (37.5)	0.274	2.01 (0.58–7.00)
**≥ 40 mg/dL**	8/22 (36.4)			14/22 (63.6)		
**Fungaemia**						
**No**	6/18 (33.3)	0.756	1.22 (0.34–4.34)	6/18 (33.3)	**0.008**	3.96 (1.44–10.88)
**Yes**	4/12 (33.3)			11/12 (91.7)		

Abbreviations: HR, Hazard ratio; CI, confidence interval; WBC, white blood cell; CFU, Colony-forming units; CSF, cerebrospinal fluid; ICP, increased intracranial pressure; CrAg, cryptococcal glucuronoxylomannan antigen.

In a multivariable regression cox model based on clinical and laboratory parameters, papilledema and cranial nerves palsies were statistically associated to mortality at weeks 2 and 10 (all p ≤ 0.02) whereas altered consciousness and fungaemia at week 10 (all p ≤ 0.02, Table 4).

**Table 4:** Cox Regression Model for death risk at weeks 2 and 10 based on clinical and laboratory parameters.

Variable	2-week mortality		10-week mortality	
	p-value	Adjusted HR (95% CI)	p-value	Adjusted HR (95% CI)
**Papilledema**	0.001	21.02 (3.31–133.44)	< 0.001	24.03 (4.97–116.21)
**Cranial nerves palsies**	0.014	8.58 (1.55–47.39)	0.001	9.48 (2.61–34.47)
**Altered consciousness**	-	-	0.023	5.07 (1.26–20.38)
**Fungaemia**	-	-	0.007	5.69 (1.61–20.10)

### Cerebrospinal fluid and sera cytokine profile and outcome

**Fig 1:**
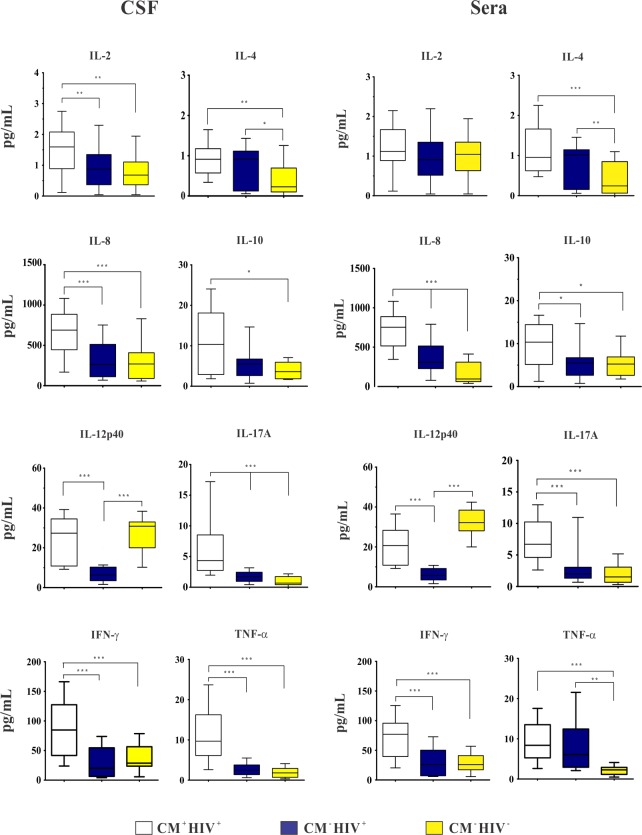
Baseline cerebrospinal fluid (CSF) and sera cytokines levels (pg/mL) of HIV-positive patients with cryptococcal meningitis (CM^+^ HIV^+^) and control groups: HIV-positive patients (CM^-^ HIV^+^) and HIV-negative subjects (CM^-^ HIV^-^). Data are shown as boxes: internal horizontal lines, medians; tops and bottom of boxes, 25th and 75th percentiles, respectively. Upper and lower bars, tenth and 90th percentiles, respectively. Statistical comparisons were made using the Kruskal-Wallis test. The symbols (*p < 0.05; ** p < 0.01; ***p < 0.001) represent the statistical analysis based on comparison of the three groups.

At admission, CM^+^ HIV^+^ patients presented CSF higher levels of IL-2, IL-4, IL-8, IL-10, IL-17A, IFN-γ and TNF-α than CM^-^ HIV^-^ ones and higher levels of IL-2, IL-8, IL-17A, IFN-γ and TNF-α than CM^-^ HIV^+^ control. Individuals with CM^-^ HIV^+^ presented higher levels of IL-4 and IL-17A than CM^-^ HIV^-^ ones. Levels of IL-17A were significantly different among the three groups (Fig. 1).

The sera cytokines median levels in CM^+^ HIV^+^ patients were higher than the controls, except IL-4 and IL-12p40 which were increased in CM^-^ HIV^+^ and CM^-^ HIV^-^, respectively. The IL-8, IL-10, IL-12p40, IL-17A and IFN-γ levels were significantly higher in CM^+^ HIV^+^ than CM^-^ HIV^+^ individuals. Also, IL-4, IL-8 and TNF-α levels were higher in CM^-^ HIV^+^ than CM^-^ HIV^-^ controls. The IL-8 levels were significantly different among the three groups (Fig. 1).

**Fig 2:**
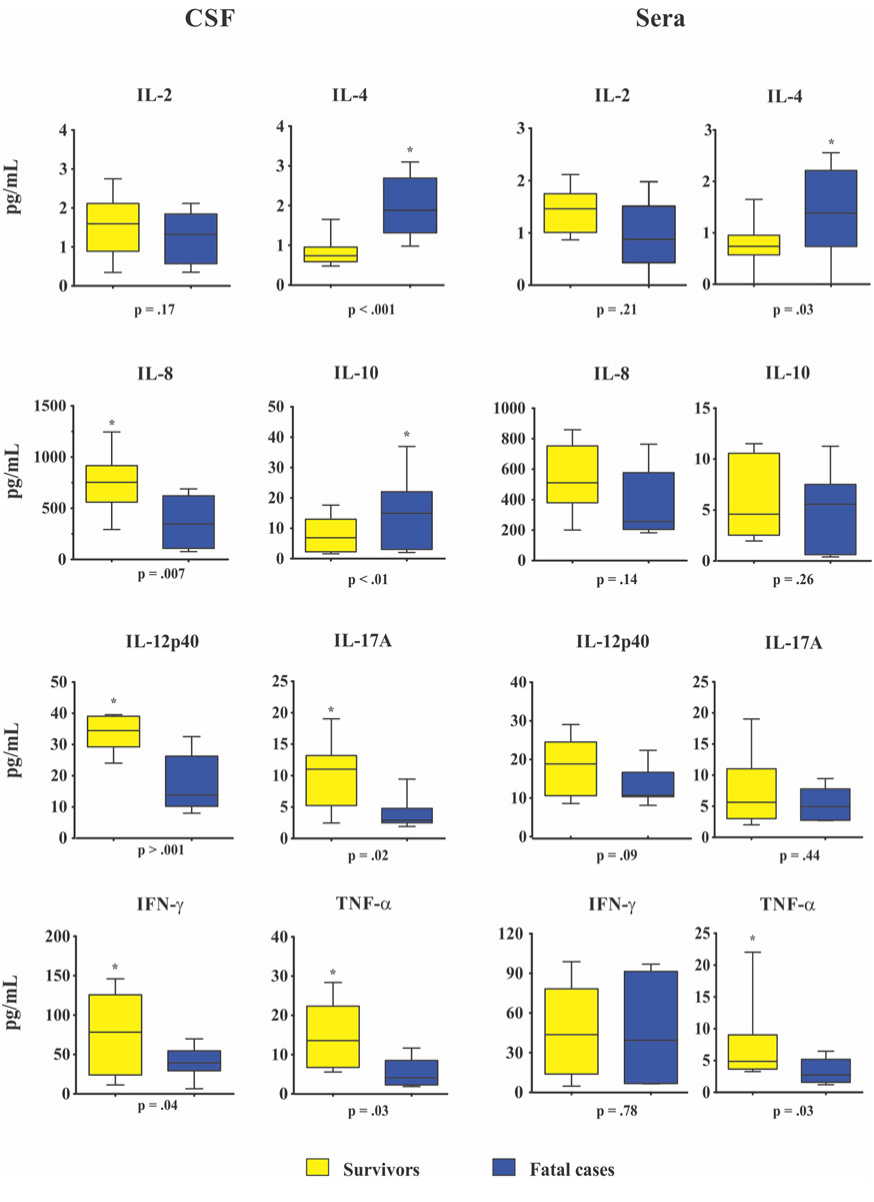
Baseline cerebrospinal fluid (CSF) and sera cytokines levels (pg/mL) in survivors (n = 20) and fatal cases (n = 10) at 2 week. Data are shown as boxes: internal horizontal lines, medians; tops and bottom of boxes, 25th and 75th percentiles, respectively. Upper and lower bars, tenth and 90th percentiles, respectively. Statistical comparisons were made using the Mann-Whitney *U* test.

The comparison between baseline CSF and sera cytokines levels of 20 survivors and 10 fatalities at week 2 showed CSF high levels of IL-8, IL-12p40, IL17-A, IFN-γ, TNF-α and higher sera TNF-α level among survivors (all p ≤ 0.04, Fig. 2). In contrast, fatal cases presented two-fold increased CSF and sera median levels of IL-4 at week 2 and 10 and two-fold increased of CSF IL-10 levels than survivors at week 2 (all p ≤ 0.03, Fig. 2). Besides, sera IL-2, IL-8, IL-10, IL-12p40, IL-17A IFN-γ and IL-2 CSF levels were not significantly different between survivors and fatal cases.

**Fig 3:**
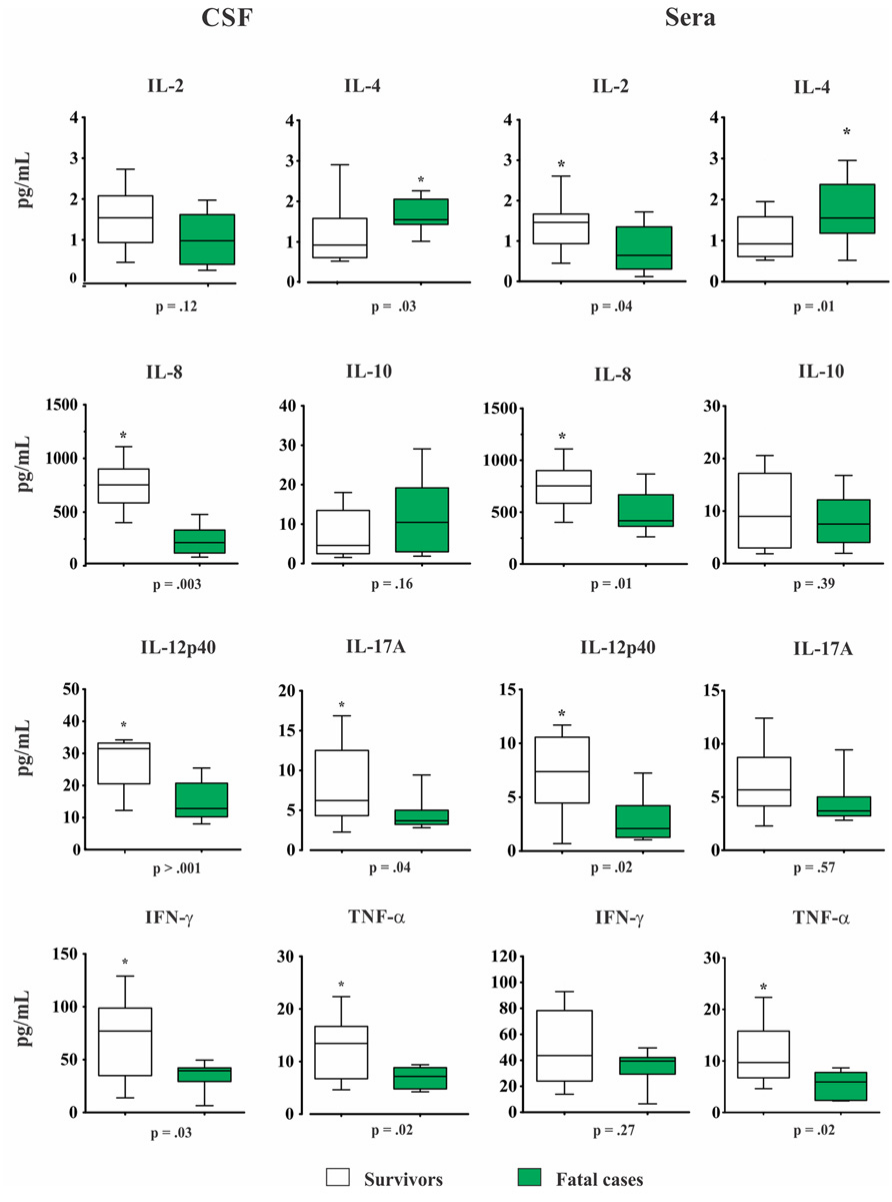
Baseline cerebrospinal fluid (CSF) and sera cytokines levels (pg/mL) in survivors (n = 13) and fatal cases (n = 17) at 10 weeks. Data are shown as boxes: internal horizontal lines, medians; tops and bottom of boxes, 25th and 75th percentiles, respectively. Upper and lower bars, tenth and 90th percentiles, respectively. Statistical comparisons were made using the Mann-Whitney *U* test.

Overall, CM patients who survived through week 10 had higher baseline CSF levels of: IL-8, IL-12p40, IL-17A, IFN-γ and TNF-α (all p ≤ 0.04, Fig. 3). Likewise, IL-2, IL-8, IL-12p40 and TNF-α high sera levels were also associated with higher survival rate at week 10, whereas a two-fold increased levels of IL-4 was associated to a poor outcome (all p ≤ 0.04, Fig. 3). In addition, patients with fungaemia presented higher IL-10 and lower IL-12p40/IL-10 sera levels (p = 0.046 and 0.039, respectively).

**Fig 4:**
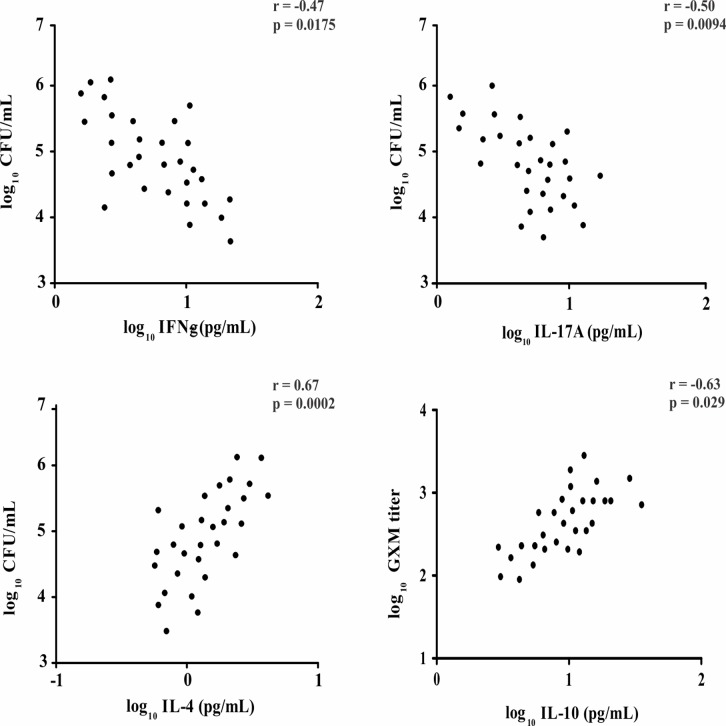
Correlation between CSF levels of IFN-γ, IL-17A, IL-4 and CSF CFU at baseline. Correlation between CSF levels of IL-10 and GXM titer. Pearson’s correlation coefficient.

Individuals with CSF persistent positive culture at week 2 presented higher baseline levels of IL-4 and IL-10, as well as lower levels of IFN-γ, IL-12p40 and IL-17A (all p ≤ 0.05). Conversely, those with CSF negative culture at week 2 exhibited high levels of IFN-γ, IL-8 and IL-17A at admission (all p ≤ 0.04). The IFN-γ and IL-17A were negatively correlated with the CFU/mL, while IL-4 and IL-10 were positively correlated with high fungal burden and CrAg (Fig. 4).

## Discussion

The epidemiological, clinical and outcome profile of patients with CM associated to AIDS herein described resembled that observed in other Brazilian, Latin America and sub-Saharan Africa regions [8,12,34,35]. Most of them were adult male, median age, presenting late HIV diagnosis and severe and disseminated fungal disease at admission [15,36,37].

In CM patients, increased ICP leads to papilledema, vomits and can contribute to altered consciousness status at admission which have been correlated to a poor outcome at weeks 2 and 10 as evidenced in most patients of this study [1,3,16,27]. The increased ICP occurs as a consequence of CSF outflow obstruction caused by the inflammatory reaction, with yeasts and/or CrAg accumulation at the arachnoid villi and subarachnoid spaces [27,38]. This fact can support the positive correlation between fungal burden with increased ICP observed in these cases [14–16,27,38]. According with several authors, mortality within 2 weeks on antifungal therapy is closely related to cryptococcal infection, different from that occurring after this period which is more related to other HIV complications [12,16].

The interaction between *Cryptococcus* spp. and the host’s immune system is a major determinant for the outcome of the disease [39,40]. In the last decades, several studies have demonstrated the protective effect of proinflammatory cytokines during clinical and experimental cryptococcal infection and its potential as adjuvant for immunotherapy [14,23,41,42]. However, clinical studies correlating the baseline cytokine profiles with fungal burden and clinical outcome are scarce and this issue needs to be better elucidated [14,16].

Cell-mediated immunity and its related cytokines are the most important arm against *C*. *neoformans* and during HIV infection, patients exhibit a switch towards a Th2 response inhibiting cellular immune response evidenced by IL-12 and IFN-γ lower levels, as well as, IL-4 and IL-10 increased levels which are associated with progression to AIDS [43–46].

The polysaccharide capsule of *Cryptococcus* spp. is considered its major virulence factor by inducting IL-10 secretion by mononuclear cells which facilitates the evasion of host defenses, inhibits the TNF-α and IFN-γ production and favors the paucity of leukocytes leading to unchecked proliferation of yeast cells [25,47–49]. Furthermore, it enhances the infectivity of HIV increasing the affinity of gp120 binding to the CD4^+^ receptor [50]. The CSF IL-10 high levels and significantly lower rate ratios of IFN-γ/IL-10 and IL-2/IL-10 observed among CM patients suggest Th1 dysregulation is pivotal and may contribute to CM pathogenesis in AIDS patients similar to that described in transplanted patients **​**and those with idiopathic CD4 lymphopenia [42,51].

Different from immunocompetent individuals or those with other T cell immune deficiencies, AIDS patients with CM often present a CSF minimal cellularity related to advanced immunossupression, as corroborated by the CD4^+^ T cells baseline count < 100 cells/mm^3^ observed in 76.7% of individuals [4,10,15]. Otherwise, most of them exhibited CSF IL-8 high levels which would favor the leukocyte migration through the blood-brain barrier. However, this action is avoided by circulating CrAg through shedding L-selectin from leukocytes surface impeding them to bind to endothelial cells and consequently to reach the CNS [24,52]. This feature is in line with previous studies in which the lack of correlation between CSF cellularity and baseline cytokine levels suggests these mediators are locally produced by activated resident cells as microglia, astrocytes and immunoregulatory NK, independently of the attracted mononuclear cells [39,40].

Cerebrospinal fluid cytokines levels and fungal load were evaluated at admission and related to outcome at weeks 2 and 10 on therapy. Survivor patients significantly presented higher CSF and sera baseline levels of TNF-α, IFN-γ and IL-8 confirming results of previous studies [14,16,41,53,54]. In addition, both IFN-γ and IL-17A levels were negatively correlated with the CSF baseline CFU count remarking their relevance to the infection control [23,29,55]. Otherwise the relatively high levels of IFN-γ and other proinflammatory cytokines observed in HIV-negative control patients can be related to their underlying diseases such as stroke, dementia, epilepsy as described elsewhere [56–58].

Although the role of Th17 cytokines in fungal immunity is not fully understood yet, previous studies have suggested that IL-17A production is related to the generation of protective immune response against *C*. *neoformans* and other intracellular pathogens as *Pneumocystis jirovecii* and *Mycobacterium tuberculosis* [29,59,60]. Experimentally, infected mice with *C*. *neoformans* H99γ, presented an increased production of IL-17, clearance of infection and a protective response against challenge with a wild-type strain reinfection [20]. These features reinforce the pivotal role of IFN-γ to control infection and suggest that IL-17 works together with other proinflammatory cytokines to modulate the immune response against *Cryptococcus* spp. [14,29,55].

Conversely, among fatal cases a 2-fold increase of anti-inflammatory cytokines IL-4 and IL-10 levels in both sera and CSF was positively correlated with severe and disseminated infection and high CSF fungal burden [14,19]. The IL-4 production mediated by polysaccharide capsule inhibits the differentiation of naïve CD4^+^ T cells (Th0) to Th1, and consequently INF-γ production, which stimulates the anti-cryptococcal activity in macrophages to destroy phagocyted microorganisms [46,61], whereas IL-10 inhibits lymphoproliferation, cytokine synthesis and expression of class II major histocompatibility complex molecules [42,62].

Recently, therapy based on cytokines has also been proposed as adjuvant in clinical and experimental cryptococcal meningitis [42,63–65]. The relevance of IFN-γ as inductive therapy aiming a faster fungal clearance from CSF improving the rate survival was remarked [63–65]. In addition, administration of recombinant IFN-γ improved clinical and immunological parameters and potentiated the effects of FLZ and AmB [42,66]. This finding was validated through a randomized and controlled trial using IFN-γ together with AmB and 5-FC in AIDS patients with CM. Moreover, it was observed an increase of the rate of fungal clearance in 30% of cases who received both therapies compared with those who received conventional therapy only [64]. Furthermore, in a murine model of pulmonary cryptococcal infection, early infusion of IL-12 increased the recruitment of inflammatory cells, enhanced the activity of FLZ, prevented the dissemination and reduced the fungal burden in lungs and brain [67,68].

Most patients herein reported had an early CM diagnosis and started antifungal therapy based on AmB and half of them died at week 10 on therapy. This mortality rate is unacceptably high and paradoxal in Brazil where the ART has been freely available in the health public services since 1996. However, most of them presented late AIDS diagnosis, severe fungal infection and received suboptimal antifungal therapy which explains this figure. Despite a low number of patients evaluated it was possible to show that cytokine profile at admission is correlated with clinical and laboratory findings which suggests that these immune mediators could be considered as markers of outcome at weeks 2 and 10 in AIDS patients with cryptococcal meningitis.
